# Molecular Rearrangement of an Aza-Scorpiand Macrocycle Induced by pH: A Computational Study †

**DOI:** 10.3390/ijms17071131

**Published:** 2016-07-14

**Authors:** Jesus Vicente De Julián-Ortiz, Begoña Verdejo, Víctor Polo, Emili Besalú, Enrique García-España

**Affiliations:** 1 ProtoQSAR SL, Parc Científic, 46980 Paterna, València, Spain; 2 Departament de Química Física, Facultat de Farmàcia, Universitat de València, Av V. Andrés Estellés 0, 46100 Burjassot, València, Spain; 3 Institut de Ciència Molecular, Universitat de València, 46980 Paterna, València, Spain; begona.verdejo@uv.es (B.V.); enrique.garcia-es@uv.es (E.G.-E.); 4 Departamento de Química Física, Universidad de Zaragoza, 50009 Zaragoza, Spain; vipolo@unizar.es; 5 Institut de Química Computacional i Catàlisi, Universitat de Girona, 17003 Girona, Spain; emili.besalu@udg.edu

**Keywords:** pH controlled, supramolecular chemistry, synthetic receptors, aza-scorpiands, semi-empirical, Density Functional Theory, Monte Carlo Multiple Minimum

## Abstract

Rearrangements and their control are a hot topic in supramolecular chemistry due to the possibilities that these phenomena open in the design of synthetic receptors and molecular machines. Macrocycle aza-scorpiands constitute an interesting system that can reorganize their spatial structure depending on pH variations or the presence of metal cations. In this study, the relative stabilities of these conformations were predicted computationally by semi-empirical and density functional theory approximations, and the reorganization from closed to open conformations was simulated by using the Monte Carlo multiple minimum method.

## 1. Introduction

The possibility of controlling the conformation of chemical structures is interesting because it opens the possibility of creating molecular machines and synthetic receptors that react to the desired stimuli [1]. Among the molecular structures showing such properties, the aza-macrocycles stand out because they show a coordinating tail, known as a scorpiand [2,3]. These systems merit attention due to their potential biological and pharmacological applications, since their ability to recognize hydrophilic and hydrophobic amino acids has been demonstrated in our laboratory [4]. Receptors for amino acid sensing [5] or drug delivery [6] are two fields for their potential applications.

We have observed that changes in the protonation state are able to induce conformational reorganizations in scorpiand-like ligands [7,8]. It has been observed by others that the presence of metal centers produces similar effects. Also, the metal coordination is influenced by the protonation state because of electrostatic repulsion [2].

A preliminary version of the present paper can be found in Internet [9]. 

**Figure 1:**
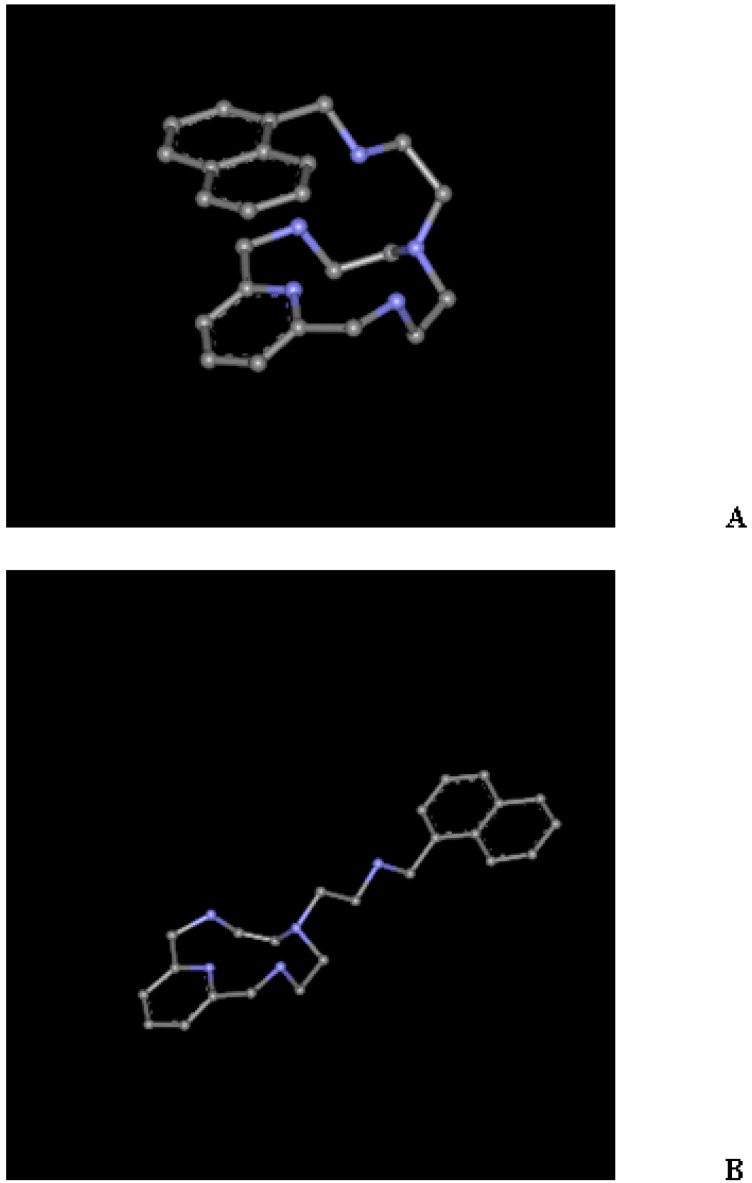
Conformations closed (**A**) and open (**B**) of the aza-scorpiand macrocycle studied.

**Figure 2:**
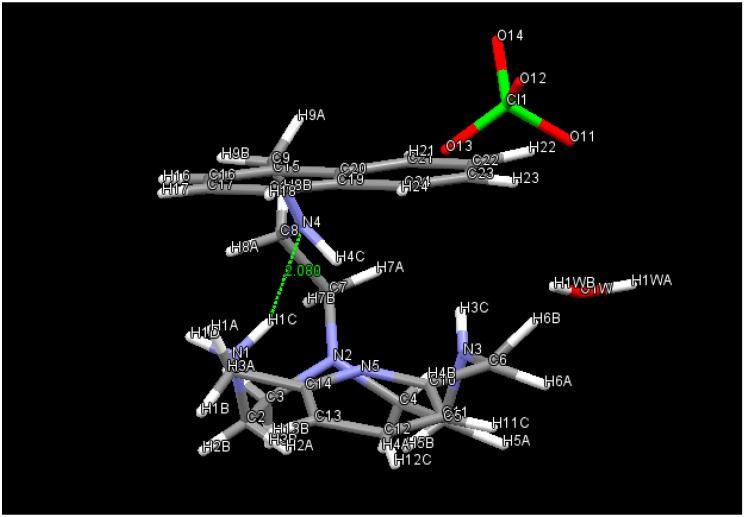
Distance between NH and N, which proves the presence of the H–bond in the monoprotonated **A**.

The simplified structures presented in Figure 1 were taken from X-ray geometries. Experimental values of the energy required to change the conformations from closed (**A**) to open (**B**) and vice versa, depending on the protonation state, are lacking. Conformation **A** is preferred for the neutral and monoprotonated species. H–bond and π–π stacking contribute to this stabilization. For instance, Figure 2 shows one of the possible H–bonds, depending on the orientation of the covalent N–H bonds, that stabilizes **A**; That is, a N1H···N4 2.08 Å distance [8]. The π–π stacking is evidenced by the minimal distance from the pyridine ring to the naphtalene ring equal to 3.26 Å [10].

The corresponding Crystallographic Information Files (CIF) can be found in the Supplementary Materials of Reference [8].

## 2. Results and Discussion

### 2.1. Conformational Search

In this study, a simulation was performed in which the pH variation induces a structure change from the so-called ‘closed’ to ‘open’ conformations.

Experimentally, it is observed that as the pH in the solution decreases, the two macrocyclic secondary amines are first protonated, and then the pendant arm’s secondary amine is also protonated. Thus, the H-bond attraction between the pendant arm and the macrocyclic amines and the attraction with the pyridine nitrogen in **A** are progressively strengthening as the protonation increases, but the electrostatic repulsion increases more sharply. This makes conformation **B** more stable for the triprotonated species. To have an understanding of the conformational stabilities involved in this system and to model its changes, the following simulation was undertaken.

Preserving conformation **A** as the initial one, the molecule structure was passed from monoprotonated to triprotonated. The conformational search was performed by the Monte Carlo multiple minimum (MCMM) method [11,12,13] with the MM+ force field [14]. The free rotation of the dihedral angles comprising the pendant arm that joins the two rings (tail) was allowed. This method finds the lowest energy conformations of a molecule by randomly varying specified dihedral angles to generate new starting conformations. These were then minimized.

MCMM was preferred to molecular dynamics (MD) trajectories because the conformational changes pursued were mainly torsions of the pendant arm atoms, and only MCMM gives these in a straightforward manner. Furthermore, the groups attached to the extremes of the pendant arm were too large to switch efficiently under MD. This last method is useful to obtain all the possible conformations in a molecule much smaller than the one studied here, or to study thermodynamic properties in molecular ensembles. An example can be the study of chemical structures surrounded by water [15,16].

**Figure 3:**
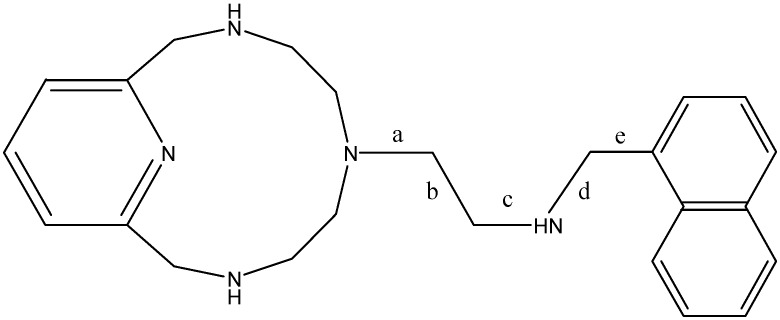
Labels for the dihedral angles in the pendant arm.

Figure 3 shows the neutral chemical structure with the nomenclature used for the different dihedral angles that have been rotated. The labels point out the central bond that undergoes torsion for each dihedron.

The conformation reorganization between **A** and **B** can be done in several ways. It can be done by the rotation of the bond between the two carbon atoms in the pendant arm, ‘b’, and the consecutive bond that joins to the 12-ring amine ‘a’. Alternatively, it can be done by inverting the 12-ring amine and rotating ‘b’ and ‘e’. Thus, if we track the changes, we see that the sequence of the torsion angles is not linear, but is even corkscrewing in some cases. These are the changes that must overcome the main rotational barriers, since all the dihedral angles suffer minor changes to obtain **B**, due to the energy minimization.

Starting from conformation **A**, it was triprotonated. MCMM simulations were performed by allowing different possible rotations in different runs.

These calculations were first attempted with the molecule in a water constant-density periodic box (standard water molecules TIP3P, equilibrated at 300 K and 1 atm, minimum distance between solvent and solute atoms: 2.3 Å), but the results failed to converge. This can be due to the fact that triprotonated conformation **A** is too far from the equilibrium, the total system with solvation water is too complex, and the dihedral torsions are too sudden to quickly balance the hydration sphere. 

For this reason, MCMM simulations were run in vacuum and the final conformations achieved were minimized in the referred periodic box boundary conditions.

**Figure 4:**
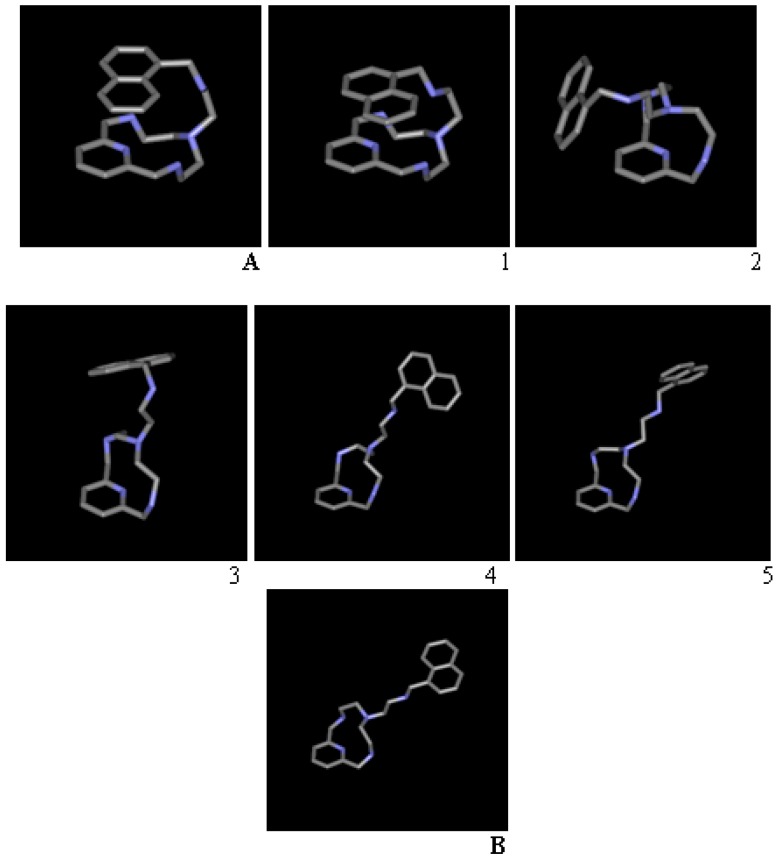
(**A**,**B**) Conformations from X-ray minimized in the periodic box. **1**–**5**, conformations obtained from MCMM in vacuum and further minimization in standard water density-constant TI3P box until their respective local minima, for different runs.

It is possible to order the resulting conformations obtained and figure a move in which **B** is obtained from **A** in successive steps, which represent relative energy minima. Table 1 and Figure 4 display different conformations obtained as local minima, all of them starting from **A**, with their respective values for the torsion angles. As said, these conformations result from MCMM and minimization within their corresponding water periodic boxes. For clarity, water molecules have been removed from Figure 4. Table 2 shows the parameters involved in the periodic box boundary step. These five structures are computationally accessible examples of intermediates, representing local minima. There is no evidence that the route shown is the most probable, and there is no experimental confirmation for each structure. The simulation methods allow us to figure out these kinds of processes for which we have only the starting and final points.

The conformations for the 12-ring obtained with MM+ are distorted with respect to the X-ray diffraction model, as well as the obtained in the Density Functional Theory (DFT) simulations. Furthermore, it seems that ring torsion is not well achieved for macrocycles, with the algorithms currently implemented in MCMM for ring inversion. It can be due to deficiences in the MM+ parameters, which were not optimized for macrocyles. In spite of these drawbacks, conformation 5, near **B**, is obtained and the sequence can be illustrative of the conformation rearrange.

Unfortunately, it was not possible to obtain a conformation near **A** from **B** with MCMM, probably due to the huge number of freedom degrees involved and the underestimation of H–bond interaction in MM+ which implied calculation times beyond our possibilities. (The entropy of **B** must be the greatest.)

### 2.2. Comparison of Total Energies

In this section, theoretical calculations were performed to predict the relative stability of each species.

Preliminary calculations to compare the total energy differences between **A** and **B** were performed by the semi-empirical methods PM3 and PM7. The results of ‘total energy’ with these methods show (Table 3) that the monoprotonated species is more stable in the closed **A** conformation, while the triprotonated is more stable in the open **B** conformation. The method PM3 performs better in this case, since the difference of the energies for the monoprotonated species calculated by PM7 should not justify the observed predominance of **A** in solution at a less acidic pH. This can be due to the adjustment of the parameters in PM7, which has been optimized for biomolecules. 

DFT calculations were tried by using different exchange functionals and basis sets. The water environment was simulated by the COSMO method. Relativistic contributions were not considered significant because no heavy nucleus was involved. The determinant importance of hydrogen bonds in the conformational equilibrium was made necessary using several polarization functions. For this reason, BP86/TZP and GGA-BP/TZ3P did not give good results, and the all-electron QZ4P basis was necessary. All the calculations performed assigned the correct energy order for the triprotonated species: the extended conformation **B** was found more stable. However, this result was also found with the monoprotonated stage when QZ4P was not used. Local Density Approximation (LDA) was useful to estimate properties in big molecules and it was the only method that allowed good results within a reasonable calculation time (ca. eight hours with a Pentium 4 @ 3.2 GHz running on Windows XP, 1 GB RAM). Although the predicted values for atomization energies estimated are maybe not accurate, the method qualitatively predicted the experimental results. Table 3 shows the results obtained. The integration accuracy was four decimal places. Three decimal places were also tried to see the influence in the final result. It was seen that the dispersion of the result was lower than the differences between the values to be compared. The conclusions were, thus, unchanged.

The difference of energy for the two conformations of the triprotonated species obtained with the method LDA/QZ4P was not enough to justify the predominance of **B** in solution at a more acid pH.

In order to have a confirmation of the trends obtained with a more complex functional, GGA-BP/TZ2P was used for a simulation without solvent.

All calculations were performed to compare atomization energies of the triprotonated species in its crystal conformation, **B**-open, and in **A**-closed. The integration accuracy was fixed in three decimal places. The calculation time for each process was approximately eight days using the same computer as before. These gave a more reliable stability prediction at the greatest protonation state. The results are displayed in Table 3.

The optimized conformations obtained were compared with the crystal ones. Using the Carbó index measured through Coulomb integrals and the local Newton-Raphson as a superposition algorithm, the similarity was quantified [17]. Thus, monoprotonated **A** and its minimized conformation obtained by LDA/QZ4P showed a Carbó index equal to 0.932, and triprotonated **B** and its respective optimization gave 0.964.

## 3. Methods

### 3.1. Monte Carlo Multiple Minimum

The MCMM method with the MM+ force field was used by allowing the free change of the dihedral angles comprised in the tail. Low-energy unique conformations were stored while high-energy or duplicate structures were discarded. The energy cut-off for discarding repeated structures was set to 6 kcal/mol. Conversely, conformation **B** was monoprotonated and treated with the same procedure, to see if it was able to reach the conformation **A**. These calculations were performed with the program Hyperchem [18].

### 3.2. Calculation of Energies

For semi-empirical methods, the Hyperchem [18] implementation of PM3 and MOPAC [19] PM7 were used.

In order to provide computationally derived relative stability estimates to have an understanding of the conformational energies, density functional theory (DFT) calculations were undertaken. The protonation states mono and tri were combined with the two possible conformational states **A** and **B** giving four chemical structures. For each protonation, the conformation energies of optimized **A** and **B** were compared to see which one would be more stable into simulated water environment. It was considered the atomisation energy, this is, the stabilization of the molecule relative to the free constitutive atoms. Several combinations of exchange functionals and basis sets were tested. The method finally chosen was all-electron local density approximation (LDA) [20] with the Vosko Wilk Nusair (VWN) functional and the Slater basis valence quadruple zeta with four polarization functions (ZORA/QZ4P), under the restricted spin formalism. Water environment was simulated by the method COSMO [21,22]. Some results also presented in this paper were obtained with the “generalized gradient approximation Becke88 Perdew86” [23] (GGA-BP) gradient corrected exchange functional and the TZ2P basis, with no solvent simulation. All DFT calculations were performed by means of the program Amsterdam Density Functional [24].

## 4. Conclusions

The MCMM method was useful in the present study, but some drawbacks must be pointed out. Thus, ring torsions in macrocycles do not seem well simulated. The pH-dependent opening of the scorpiand was easy to predict, but the reverse effect was not possible to simulate.

DFT was a good method to give an account of the experimental stability, although the all-electron basis was necessary. The LDA approximation with the COSMO method was enough to obtain the relative stabilities clearly according to the experiments at lower protonation. At more acidic pH, the more complex functional GGA-BP with a somewhat simple basis gave more reliable results.

The optimized conformations obtained were in accordance with the crystal ones, as verified by quantum molecular similarity.

Due to the nature of the studied molecules, the balance among intramolecular forces that determines a given conformation is due to several counteracting interactions. These make it so that pH changes induce conformation rearrangements in these species. The key aspect is that relatively weak intramolecular forces that induce a given folded conformation, such as H-bonds and π–π stacking, are balanced and eventually overcome by electrostatic forces that can induce a conformation change that minimizes the intramolecular charge repulsion by extending out the structure. This process can be consistently modeled by the computational methods used here. Since the intramolecular forces involved are common to other kinds of molecular systems, the methods disclosed here could be applied to other structures, particularly to molecular systems in which electrostatic forces are balanced with staking and H–bonding.

This study points out that molecular modeling can be used to design new molecules able to reorganize their conformations due to pH variations. This opens interesting possibilities in the design of molecular systems with controllable motion.

## Figures and Tables

**Table 1:** Torsion angles (°) in the tail for different conformations obtained with MCMM, starting with conformation **A**.

Conformation	Torsions Allowed	a	b	c	d	e
**A**	-	−146.8	74.4	−170.9	80.7	77.0
1	12-ring, d	−148.4	59.1	176.8	144.9	66.3
2	12-ring, b, d	68.2	179.2	−91.2	71.3	74.7
3	a, b	−67.9	−175.5	94.2	−67.3	105.3
4	d	−62.6	−167.8	−177.3	−176.9	−814
5	12-ring, a	−154.9	170.8	177.6	172.9	−87.1
**B**	-	69.4	−176.5	−177.2	−179.2	84.6

**Table 2:** Geometric parameters involved in the water box calculations for each conformation obtained from MCMM.

Conformation	Smallest Box Enclosing Solute/Å			Cubic Periodic Box Edge/Å	Maximum Number of Water Molecules
	X	Y	Z		
**A**	6.90	6.19	7.73	18.10	216
1	7.65	5.87	7.80	18.10	216
2	8.08	6.91	9.07	18.10	216
3	7.76	5.71	10.75	21.51	329
4	7.49	5.42	15.48	30.96	980
5	7.30	5.17	19.91	31.81	1064
**B**	6.90	3.29	17.62	35.25	1447

**Table 3:** Calculated energies for each protonation state and conformation, closed (**A**) and open (**B**) ^1^.

Starting Conformation	Number of H+	Method	Environment Simulation Method	Total Energy/kcal/mol ^2^	Difference A–B/kcal/mol
**A**	1	PM3	vacuum	**−6124.84**	−9.14
**B**	1	PM3	vacuum	−6115.70	
**A**	3	PM3	vacuum	−5752.39	−14.13
**B**	3	PM3	vacuum	**−5757.88**	
**A**	1	PM7	vacuum	**−99257.16**	5.49
**B**	1	PM7	vacuum	−99257.00	
**A**	3	PM7	vacuum	−99411.69	−0.16
**B**	3	PM7	vacuum	**−99424.32**	
**A**	1	LDA/QZ4P	COSMO	**−9171.05**	−6.42
**B**	1	LDA/QZ4P	COSMO	−9162.84	
**A**	3	LDA/QZ4P	COSMO	−9133.36	12.63
**B**	3	LDA/QZ4P	COSMO	**−9134.40**	
**A**	3	GGA-BP/TZ2P	vacuum	−8396.54	−8.21
**B**	3	GGA-BP/TZ2P	vacuum	**−8408.01**	

^1^ The values of ‘total energy’ are not absolute values. They depend on the contributions that each method uses in its determination. Values obtained with different methods cannot be compared. Values obtained with one given method for monoprotonated species cannot be compared with those obtained for the triprotonated, because they have different numbers of atoms. Only energies obtained with exactly the same method for isomers or ‘conformers’ can be directly compared. Thus, the absolute value of the energy is not important. The key is the difference in such values between species with the same composition. An experimental value related to this ‘total energy’ is the heat of formation. These have not been determined experimentally at present; ^2^ The minimum energy for each pair **A** **B** is indicated in bold.
